# Supplementary material for: Genome sequencing reveals the impact of non-canonical exon inclusions in rare genetic disease
Source: medRxiv. 2024 Dec 26:2024.12.21.24318325. Preprint. [Version 1] doi: 10.1101/2024.12.21.24318325 (PMC11703292; doi:10.1101/2024.12.21.24318325)
Supplement: Supplementary file 1 [file NIHPP2024.12.21.24318325V1-supplement-1.pdf]

medRxiv preprint doi: https://doi.org/10.1101/2024.12.21.24318325; this version posted December 26, 2024. The copyright holder for this preprint (which was not certified by peer review) is the author/funder, who has granted medRxiv a license to display the preprint in perpetuity.

It is made available under a CC-BY 4.0 International license .

Supplemental Table 1. Solved/Probable solved cases

medRxiv preprint doi: https://doi.org/10.1101/2024.12.21.24318325; this version posted December 26, 2024. The copyright holder for this preprint (which was not certified by peer review) is the author/funder, who has granted medRxiv a license to display the preprint in perpetuity.

It is made available under a CC-BY 4.0 International license .

**Participant ID**	**Phenotypic Category**	**Gene**	**Variant**	**Patient Phenotype**	**Reason diagnosis was missed on previous testing**	**Associated Disorder (OMIM)**
PMGRC-13-13-0	Syndromic	*PTHLH*	c.4C>T, p.Gln2*	Pseudohypoparathyroidism, Chiari 1 malformation, shortened metacarpals	Limited Original Testing	Brachydactyly, type E2 (#613382)
PMGRC-32-32-0	Other	*FLG*	c.1297_1298del, p.Asp433fs<br>c.2282_2285del, p.Ser761fs	Eczematoid dermatitis, numerous arcuate plaques with erythematous border, perivascular and interstitial dermatitis with neutrophils on biopsy, ichthyosiform xerosis	Limited Original Testing	Ichthyosis vulgaris (#146700)
PMGRC-43-43-0	Syndromic	*HECTD1*	c.2082C>G, p.Phe694Leu	Hypotonia, oral dysphagia, macrocephaly, autism spectrum disorder, somatic overgrowth syndrome, hypothalamic obesity, tube feeding, esotropia, stereotypic movements, abnormal brain MRI, dysmorphic facial features	New Information/Reclassification/Candidate gene now linked to syndrome	HECTD1-neurodevelopmental disorder (under review)
PMGRC-46-46-0	Syndromic	*PPP1R3F*	c.554_555del, p.His185fs*66	Hypotonia, tube feeding, autism spectrum disorder, speech/language delays, developmental delay	New Information/Reclassification/Candidate gene now linked to syndrome	X-linked PPP1R3F neurodevelopmental disorder [27]
PMGRC-88-88-0	Syndromic	*NARS2*	c.749G>A, p.Arg250Gln	Critically ill neonate with multisystemic disease. A few months later, new findings of interstitial lung disease and refractory seizures	Limited Original Testing	Combined oxidative phosphorylation deficiency 24 (#616239)
PMGRC-101-101-0	Syndromic	*CBL*	c.1259G>A, p.Arg420Gln	Hemihypertrophy, nystagmus, club foot	Previously known	Noonan syndrome-like disorder with or without juvenile myelomonocytic leukemia (#613563)
PMGRC-114-114-0	Connective tissue & Musculoskeletal disorders	*ACAN*	c.4753del, p.Asp1585fs<br>c.757+4A>G	Severe scoliosis, spondyloepimetaphyseal dysplasia, genu valgum, short stature	Previously known	Spondyloepimetaphyseal dysplasia, aggrecan type (#612813)

medRxiv preprint doi: https://doi.org/10.1101/2024.12.21.24318325; this version posted December 26, 2024. The copyright holder for this preprint (which was not certified by peer review) is the author/funder, who has granted medRxiv a license to display the preprint in perpetuity.

It is made available under a CC-BY 4.0 International license .

PMGRC-124-124-0	Syndromic	*HFE*	c.187C>G, p.His63Asp<br>c.892G>A, p.Glu298Lys	Liver disease, hyperammonemia, encephalopathy, hyperbilirubinemia	Limited Original Testing	Hemochromatosis, type 1 (#235200)
PMGRC-146-146-0	Syndromic	*ARID1B*	c.3235+700C>G	Developmental delay, gross motor delay, abnormal brain MRI, mixed receptive-expressive language disorder, dysmorphic facial features	Limited Original Testing	Coffin-Siris syndrome 1 (#135900)
PMGRC-148-148-0	Syndromic	*RNU4-2*	n.64_65insT	Hypotonia, global developmental delay, speech delay, febrile seizures, optic nerve hypoplasia, tube feeding, dysmorphic facial features	Limited Original Testing	ReNU syndrome (#620851)
PMGRC-151-151-0	Syndromic	*GLMN*	c.157_161del, p.Glu52_Lys53insTer	Clinical diagnosis of glomuvenous malformations	Limited Original Testing	Glomuvenous malformations (#138000)
PMGRC-158-158-0	Syndromic	*COQ2*	c.421G>C, p.Val141Leu<br>c.138dup, p.Ala47fs	Retinal atrophy, horseshoe kidney, nephrotic syndrome	New Information/Reclassification	Coenzyme Q10 deficiency, primary, 1 (#607426)
PMGRC-170-170-0	Neurodevelopmental	*GRIA4*	c.1643G>C, p.Trp548Ser	Hypotonia, feeding difficulties, global developmental delays of motor and language skills, microcephaly, delayed visual attention, abnormal brain MRI	New Information/Reclassification	Neurodevelopmental disorder with or without seizures and gait abnormalities (#617864)
PMGRC-175-175-0	Syndromic	*PTPN11*	c.1510A>G, p.Met504Val	Pulmonary valve stenosis, toe syndactyly, widely spaced nipples	Previously Known	Noonan syndrome 1 (#163950)
PMGRC-176-176-0	Syndromic	*SCN8A*	c.2549G>A, p.Arg850Gln	Refractory seizures, epileptic encephalopathy, infantile spasms, global developmental delay, hypotonia, tube feeding, respiratory insufficiency, hypotonia	Previously Known	Developmental and epileptic encephalopathy 13 (#614558)
PMGRC-178-178-0	Neurodevelopmental	*CHD1*	c.1010C>T, p.Thr337Ile	Motor delay, hypotonia, stereotypic behaviors, autism spectrum disorder	New Information/Reclassification	Pilarowski-Bjornsson syndrome (#617682)

medRxiv preprint doi: https://doi.org/10.1101/2024.12.21.24318325; this version posted December 26, 2024. The copyright holder for this preprint (which was not certified by peer review) is the author/funder, who has granted medRxiv a license to display the preprint in perpetuity.

It is made available under a CC-BY 4.0 International license .

PMGRC-204-204-0	Neurodevelopmental	*FBXO31*	c.1000G>A, p.Asp334Asn	Abnormal brain MRI, gross motor delay, speech delay, hypertonia, lower extremity spasticity, esotropia, mixed receptive-expressive language disorder	New Information/Reclassification	FBXO31-related spastic-dystonic cerebral palsy syndrome [28]
PMGRC-205-205-0	Syndromic	*SLC6A8*	c.1255-3_1255-2delCA	Acute liver failure, acute kidney injury, autism spectrum disorder	Limited Original Testing	Cerebral creatine deficiency syndrome 1 (#300352)
PMGRC-212-212-0	Other	*OCA2*	c.1465A>G, p.Asn489Asp and a complex rearrangement with deep intronic breakpoints	Oculocutaneous albinism	Limited Original Testing	Oculocutaneous albinism, type II (#203200)
PMGRC-220-220-0	Syndromic	*HADHB*	c.1390-515_1390-499del	Hypoparathyroidism, LCHAD, mitochondrial trifunctional protein deficiency, pancytopenia, bone marrow deficiency, nephrotic syndrome, tube feeding	Limited Original Testing	Mitochondrial trifunctional protein deficiency 2 (#620300) [29]
PMGRC-265-265-0	Syndromic	*RNF220*	c.1094G>A, p.Arg365Gln	Bilateral sensorineural hearing loss, leukodystrophy, seizures, mixed receptive-expressive language disorder, photoreceptor degeneration, intention tremor	New Info/Reclassification	Leukodystrophy, hypomyelinating, 23, with ataxia, deafness, liver dysfunction, and dilated cardiomyopathy (#619688)
PMGRC-316-316-0	Syndromic	*PTPN11*	c.794G>A, p.Arg265Gln	Short stature, gross motor delays	Limited Original Testing	Noonan syndrome 1 (#163950)
PMGRC-332-332-0	Neurodevelopmental	*CRPPA*	c.789+973C>G	Global developmental delay, hypotonia, chronic lung disease, retinopathy, severe optic atrophy, tube feeding, abnormal brain MRI	Limited Original Testing	Muscular dystrophy-dystroglycanopathy (congenital with brain and eye anomalies), type A, 7 (#614643)
PMGRC-355-355-0	Neurodevelopmental	*TCF7L2*	c.1156C>T, p.Arg240Cys	Walking difficulties, unsteady gait, lateral lisps, ADHD, distal weakness, hammertoes, gynecomastia, learning disabilities	New Information/Reclassification	TCF7L2-associated syndromic neurodevelopmental disorder [30]

medRxiv preprint doi: https://doi.org/10.1101/2024.12.21.24318325; this version posted December 26, 2024. The copyright holder for this preprint (which was not certified by peer review) is the author/funder, who has granted medRxiv a license to display the preprint in perpetuity.

It is made available under a CC-BY 4.0 International license .

PMGRC-366-366-0	Neurodevelopmental	*DDX17*	c.1077G>A, p.Trp359*	Hypotonia, global developmental delay, gross motor delay, mixed receptive-expressive language disorder, foot drop, steppage gait, pes planus, ataxic gait	New Information/Reclassification	DDX17- associated neurodevelopmental disorder [31]
PMGRC-388-388-0	Syndromic	*RNU4-2*	n.64_65insT	Global developmental delay, hemiparesis, microcephaly, tube feeding, growth delays, truncal hypotonia, autism spectrum disorder, nonverbal, speech apraxia, sensory processing difficulties, abnormal brain MRI	Limited Original Testing	ReNU syndrome (#620851)
PMGRC-392-392-0	Neurodevelopmental	*COQ4*	c.718C>T, p.Arg240Cys<br>c.202+4A>C	Spastic diplegia, gross motor delays, speech articulation delay, unsteady gait, walking difficulties	New Information/Reclassification	Spastic ataxia 10, (#620666)
PMGRC-403-403-0	Syndromic	*PEX1*	c.1359+601A>G	Zellweger Spectrum Disorder, elevated very long-chain fatty acids, decreased plasmalogen	Limited Original Testing	Peroxisome biogenesis disorder 1A (#214100)
PMGRC-418-418-0	Neurodevelopmental	*ZIC2*	c.1030_1032del, p.Trp359*	Alobar holoprosencephaly	Limited Original Testing	Holoprosencephaly 5 (#609637)
PMGRC-437-437-0	Syndromic	*GLI2*	c.1111_1120del, p.Ile371fs	Septopreoptic holoprosencephaly, solitary median maxillary incisor syndrome, low thyroid, underdeveloped pituitary, sensory processing disorder, failure to thrive	Limited Original Testing	Holoprosencephaly 9 (#610829)
PMGRC-440-440-0	Syndromic	*ZIC2*	c.1013del, p.Pro338fs	Deafness, cerebral palsy, holoprosencephaly	Previous testing information not provided	Holoprosencephaly 5 (#609637)
PMGRC-451-451-0	Syndromic	*ZIC2*	c.1377_1406dup, p.Ala461_Ala470dup	Semilobar holoprosencephaly, diabetes insipidus, delayed development	Limited Original Testing	Holoprosencephaly 5 (#609637)
PMGRC-509-509-0	Syndromic	*RNU4-2*	n.64_65insT	Microcephaly, hypotonia, delayed myelination, gross motor delay, brachycephaly, abnormal brain MRI, global developmental delay	Limited Original Testing	ReNU syndrome (#620851)
PMGRC-538-538-0	Syndromic	*AGO2*	c.602G>T, p.Gly201Val	Hip dysplasia, congenital hypotonia, cleft palate, global developmental delay, hearing loss, Pierre Robin sequence, seizures, speech delay, cerebral palsy,	New Information/Reclassification	Lessel-Kreienkamp syndrome (#619149)

medRxiv preprint doi: https://doi.org/10.1101/2024.12.21.24318325; this version posted December 26, 2024. The copyright holder for this preprint (which was not certified by peer review) is the author/funder, who has granted medRxiv a license to display the preprint in perpetuity.
It is made available under a CC-BY 4.0 International license .

				scoliosis, congenital heart defects, dysmorphic facial features		
PMGRC-540-540-0	Syndromic	*KMT2C*	c.5668C>T, p.Arg1890*	Hypotonia, fine motor delay, gross motor delay, sensory processing disorder, speech delay, decreased oral tone, selective mutism, ADHD, developmental coordination disorder, dysmorphic facial features	Limited Original Testing	Kleefstra syndrome 2 (#617768)
PMGRC-569-569-0	Neurodevelopmental	*STAG2*	87kb deletion of chrX:123,946,386-124,003590<br>Exon 1-4 deletion	Holoprosencephaly, global developmental delay, scoliosis, apraxia of speech, vision problems, vertebral anomaly	Previously Known	Holoprosencephaly-13, X-linked (#301043)
PMGRC-599-599-0	Neurodevelopmental	*RNU4-2*	n.69C>T	Small for gestational age, abnormal visual development/eye movement irregularity, global developmental delay, hypotonia, abnormal brain MRI	Limited Original Testing	ReNU syndrome (#620851)
PMGRC-635-635-0	Syndromic	*CDK13*	c.425dup, p.Leu143fs	Congenital heart disease, dysmorphic facial features, short stature, ADHD, global developmental delay	New Information/Reclassification	Congenital heart defects, dysmorphic facial features, and intellectual developmental disorder (#617360)
PMGRC-658-658-0	Syndromic	*DBT*	c.1282-4218G>A	Prenatal diagnosis of Argininosuccinic acid lyase deficiency and thiamine-responsive maple syrup urine disease	Limited Original Testing	Argininosuccinic aciduria, Maple syrup urine disease, type II (#620699)
PMGRC-677-677-0	Syndromic	*TPM3*	c.835C>G, p.Leu279Val	Pierre Robin sequence, tube feeding, gastroparesis, mannose-binding lectin deficiency, recurrent aspiration pneumonia and sinusitis, abnormal brain MRI, hypotonia	Limited Original Testing	Congenital Myopathy 4A, Autosomal Dominant (#255310)
PMGRC-700-700-0	Syndromic	*OFD1*	c.2413dup, p.Gln805fs	Semilobar holoprosencephaly, cleft lip and palate, bilateral postaxial polydactyly of hands and feet, bilateral cryptorchidism, Dandy-Walker malformation, ventriculomegaly, hypotelorism, seizures	Limited Original Testing	Joubert syndrome 10 (#300804)

medRxiv preprint doi: https://doi.org/10.1101/2024.12.21.24318325; this version posted December 26, 2024. The copyright holder for this preprint (which was not certified by peer review) is the author/funder, who has granted medRxiv a license to display the preprint in perpetuity.

It is made available under a CC-BY 4.0 International license .

PMGRC-753-753-0	Syndromic	*MSL2*	c.535G>T, p.Glu179*	Gross motor delay, speech delay, hypotonia, microcephaly, frontal bossing, fine hair, ptosis, pectus excavatum, low muscle tone, Mongolian spot in lower back, global developmental delay	New Information/Reclassification	Karayol-Borroto-Haghshenas neurodevelopmental syndrome (#620985)
PMGRC-812-812-0	Syndromic	*NDST1*	c.1850 C>T, p.Thr617Ile	Ankyloglossia, gross motor delays, thoracolumbar kyphosis, global developmental delay, axial hypotonia	New Information/Reclassification	Intellectual Developmental Disorder, Autosomal Recessive 46 (#616116)
PMGRC-817-817-0	Syndromic	*MT-TL1*	n.14A>G (m.3243A>G)	Fatigue, headache, recurrent fever, tinnitus, recurrent episodes of infection, sudden loss of hearing	Limited Original Testing	Diabetes-deafness syndrome (#520000)
PMGRC-890-890-0	Cardiovascular	*TNN*	c.59604_59607delAAAG p.Gly19871fs	Dilated Cardiomyopathy	Limited Original Testing	Dilated Cardiomyopathy, 1G (#604145)
PMGRC-897-897-0	Cardiovascular	*SCN5A*	c.3911C>T, p.Thr1304Met	Dilated Cardiomyopathy	Previously Known	Dilated Cardiomyopathy, 1E (#601154)
PMGRC-904-904-0	Cardiovascular	*DMD*	c.9851G>A, p.Trp3284*	Dilated Cardiomyopathy	Previously Known	Dilated Cardiomyopathy, 3B (#302045)
PMGRC-911-911-0	Cardiovascular	*MYH6*	c.2489C>T, p.Pro830Leu	Dilated Cardiomyopathy	Limited Original Testing	Dilated Cardiomyopathy, 1EE (#613252)
PMGRC-914-914-0	Cardiovascular	*TNNT2*	c.517C>T, p.Arg173Trp	Dilated Cardiomyopathy	Limited Original Testing	Dilated Cardiomyopathy, 1D (#601494)
PMGRC-917-917-0	Cardiovascular	*RBM20*	c.2497dupA, p.Arg833fs	Dilated Cardiomyopathy	Limited Original Testing	Dilated Cardiomyopathy, 1DD (#613172)
PMGRC-921-921-0	Cardiovascular	*TNNI3K*	c.2302G>A, p.Glu768Lys	Dilated Cardiomyopathy	Limited Original Testing	Cardiac conduction disease with or without dilated cardiomyopathy (#616117)

medRxiv preprint doi: https://doi.org/10.1101/2024.12.21.24318325; this version posted December 26, 2024. The copyright holder for this preprint (which was not certified by peer review) is the author/funder, who has granted medRxiv a license to display the preprint in perpetuity.

It is made available under a CC-BY 4.0 International license .

PMGRC-991-991-0	Connective tissue & Musculoskeletal disorders	*TGFBR1*	c.1355del, p.Pro452fs	Thoracic aortic aneurysm, Aortic dissection	Previous testing information not provided	Loeys-Dietz syndrome 1 (#609192)
PMGRC-1040-1040-0	Connective tissue & Musculoskeletal disorders	*FBN1*	c.4096G>A, p.Glu1366Lys	Thoracic aortic aneurysm, Aortic dissection	Previous testing information not provided	Marfan syndrome (#154700)
PMGRC-1091-1091-0	Syndromic	*CREBBP*	exon 2 deletion	Failure to thrive, global developmental delay, pectus excavatum, axial hypotonia, abnormal brain MRI, dysmorphic features	Limited Original Testing	Rubinstein-Taybi syndrome 1 (#180849)

ADHD attention deficit hyperactivity disorder, *LCHAD* long-chain 3-hydroxyacyl-CoA dehydrogenase enzyme deficiency, *MRI* magnetic resonance imaging

medRxiv preprint doi: https://doi.org/10.1101/2024.12.21.24318325; this version posted December 26, 2024. The copyright holder for this preprint (which was not certified by peer review) is the author/funder, who has granted medRxiv a license to display the preprint in perpetuity.

It is made available under a CC-BY 4.0 International license .

Supplemental Table 2. Candidate genes identified in unsolved cases. All genes are deposited in GeneMatcher.

**Participant ID**	**Candidate Gene**	**Variant**
PMGRC-86-86-0	*NRXN2*	c.2107G>A, p.Gly703Ser
PMGRC-104-104-0	*GALNT16*	c.502+1G>A
PMGRC-116-116-0	*ARHGAP21; PDPK1*	c.3692_3693del;<br>c.1588G>A, p.Gly530Arg
PMGRC-137-137-0	*CSMD3*	c.3158dup, p.Arg1530*
PMGRC-217-217-0	*SLC16A13; HIVEP1; MHY7*	c.410G>A, p.Arg137Gln,<br>c.395A>G, p.Tyr132Cys;<br>c.6212dupA, p.Tyr2071*;<br>c.4187G>T, p.Arg1396Leu
PMGRC-239-239-0	*FGD5*	c.3372_3373del,<br>p.Gly1125Glufs*54
PMGRC-255-255-0	*NRXN2; STRN3*	c.2605C>T, p.Arg869Trp;<br>c.1875C>A, p.Tyr625*
PMGRC-279-279-0	*VWA3B*	c.59G>T, p.Gly20Val<br>c.1737+1G>A
PMGRC-291-291-0	*NSL1*	c.314-2_315delAGAT
PMGRC-312-312-0	*TJP1*	c.3927-2_3927-1del
PMGRC-363-363-0	*CEP350*	c.2258del, p.Gly753Glufs*11
PMGRC-423-423-0	*FAM193A; MEX3D; CLPTM1*	c.1390-2A>G;<br>c.595+1G>C;<br>c.953A>G, p.Tyr318Cys
PMGRC-446-446-0	*TP53BP2*	c.40G>A, p.Val14Met<br>c.3240del, p.Asp1080Glufs*5
PMGRC-485-485-0	*HEATR1; PRR30*	c.3613C>T, p.Gln1205*;<br>c.275del, p.Pro92Glnfs*33, c.-365-6C>T
PMGRC-525-525-0	*PLXNC1*	c.416del, p.Leu139Argfs*4
PMGRC-746-746-0	*DOP1A; PCDH9*	c.3559dup, p.Ile1187Asnfs*5;<br>c.3291del, p.Ser1098Leufs*25

medRxiv preprint doi: https://doi.org/10.1101/2024.12.21.24318325; this version posted December 26, 2024. The copyright holder for this preprint (which was not certified by peer review) is the author/funder, who has granted medRxiv a license to display the preprint in perpetuity.

It is made available under a CC-BY 4.0 International license .

PMGRC-772-772-0	*MUC5B*	c.15477+1G>C
PMGRC-782-782-0	*CD177*	c.99G>A, p.Trp33*
PMGRC-832-832-0	*TLN2; IL9*	c.4645C>T, p.Asp1549Tyr; c.183+6T>C, p.Ser893Arg, c.202T>C, p.Cys68Arg
PMGRC-949-949-0	*GNRH2*	c.213_214insTGTC
PMGRC-985-985-0	*FMNL1*	c.1379del, p.Pro460Glnfs*19
PMGRC-992-992-0	*PRR14L*	c.4166del, p.Tyr318Cys
PMGRC-999-999-0	*SLC17A9*	c.287C>G, p.Tyr132Cys
PMGRC-1017-1017-0	*NXF3*	c.1231G>T, p.Glu411*
PMGRC-1030-1030-0	*CORO1C*	c.474G>A, p.Trp158*
PMGRC-1048-1048-0	*NEURL4*	c.1606G>T, p.Glu536*
PMGRC-1050-1050-0	*CUL1*	c.571_575del, p.Arg191Trpfs*2
