# Supplementary material for: Genome sequencing reveals the impact of non-canonical exon inclusions in rare genetic disease
Source: medRxiv. 2024 Dec 26:2024.12.21.24318325. Preprint. [Version 1] doi: 10.1101/2024.12.21.24318325 (PMC11703292; doi:10.1101/2024.12.21.24318325)
Supplement: Supplementary file 2 [file media-1.pdf]

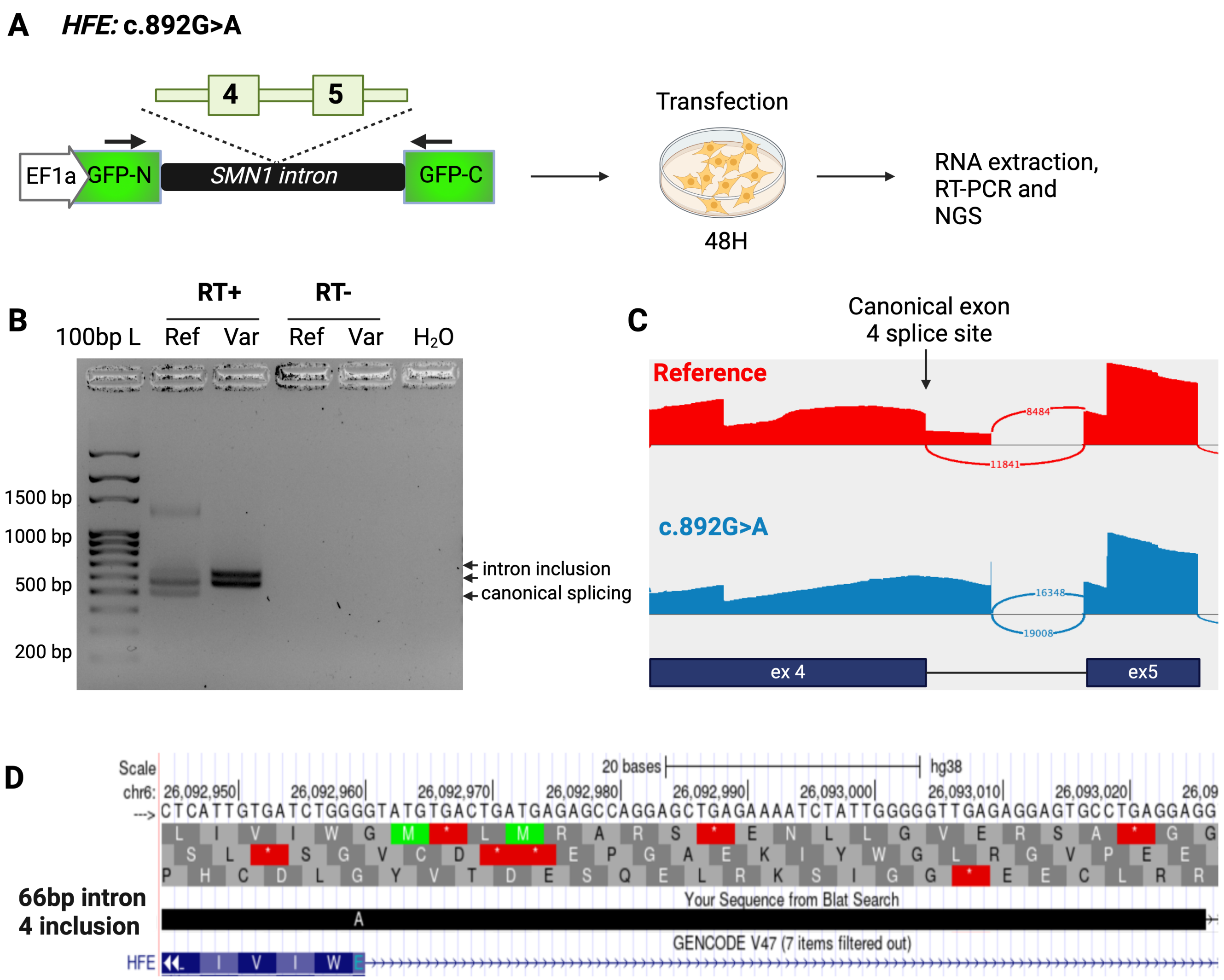

Supplemental Figure 1. Mini-gene splicing assay for the *HFE* c.892G>A variant. A) *HFE* exon 4 and 5 reference and c.892G>A variant constructs were cloned into a mini-gene split *GFP* construct, in which N- and C-terminal parts of the *GFP* gene were separated by *SMN1* introns 7 and 8 (NM_000344). The construct was expressed in HEK293 cells for 48 hours followed by mRNA extraction, cDNA generation, PCR and NGS amplicon sequencing. Black arrows on the construct image indicate primer placement. **B)** RT-PCR gel electrophoresis demonstrates canonical and alternative splicing event for the reference sequence and two alternative splicing events for the variant sequence. **C)** Sashimi plot demonstrates normal splicing between exon 4 and 5 as well as a 66 bp intron inclusion event in intron 4. Variant allele does not show any normal splicing between exons 4 and 5 and exclusively shows the 66 bp inclusion after exon 4. **D)** The intronic inclusion is predicted to introduce a premature stop-gain leading to nonsense-mediated decay of this allele.
