# Supplementary material for: Genome sequencing reveals the impact of non-canonical exon inclusions in rare genetic disease
Source: medRxiv. 2024 Dec 26:2024.12.21.24318325. Preprint. [Version 1] doi: 10.1101/2024.12.21.24318325 (PMC11703292; doi:10.1101/2024.12.21.24318325)
Supplement: Supplementary file 3 [file media-2.pdf]

PMGRC-146-146-0

c.3235+700C>G

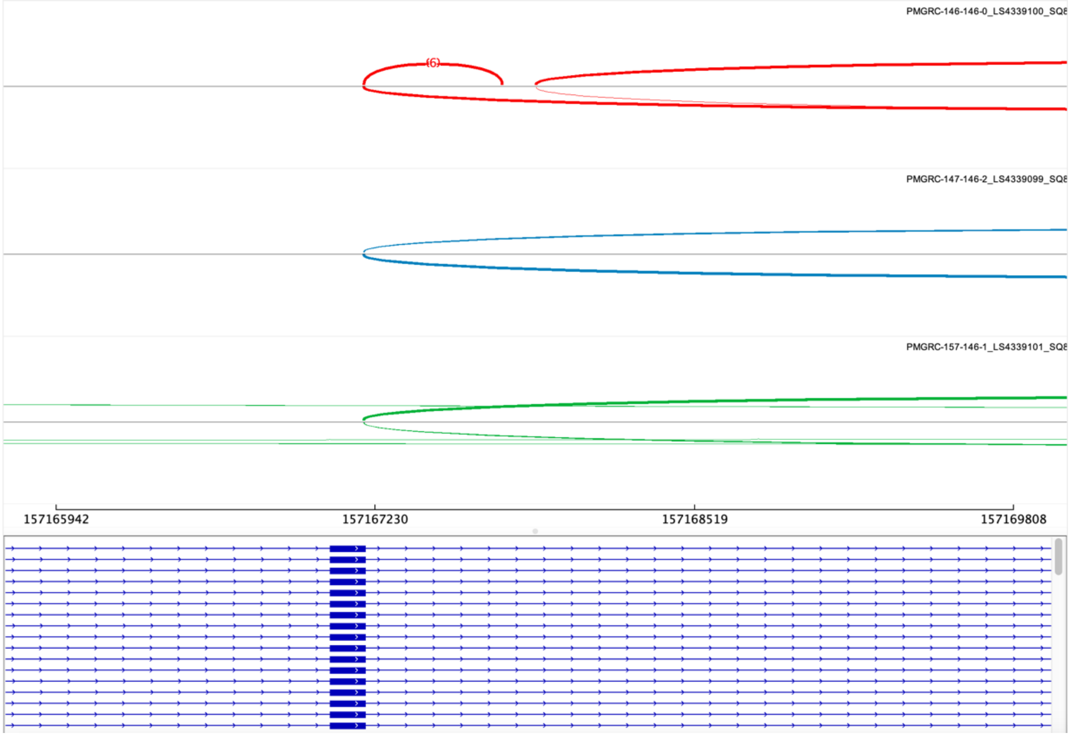

PMGRC-332-332-0

c.789+973C>G

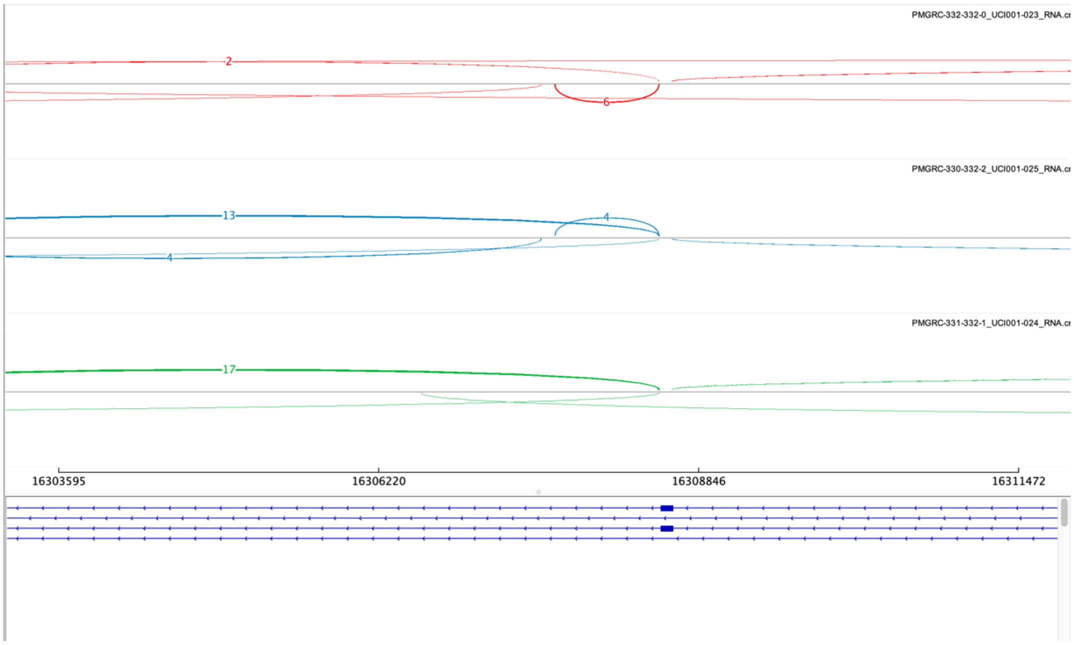

PMGRC-403-403-0

c.1359+601A>G

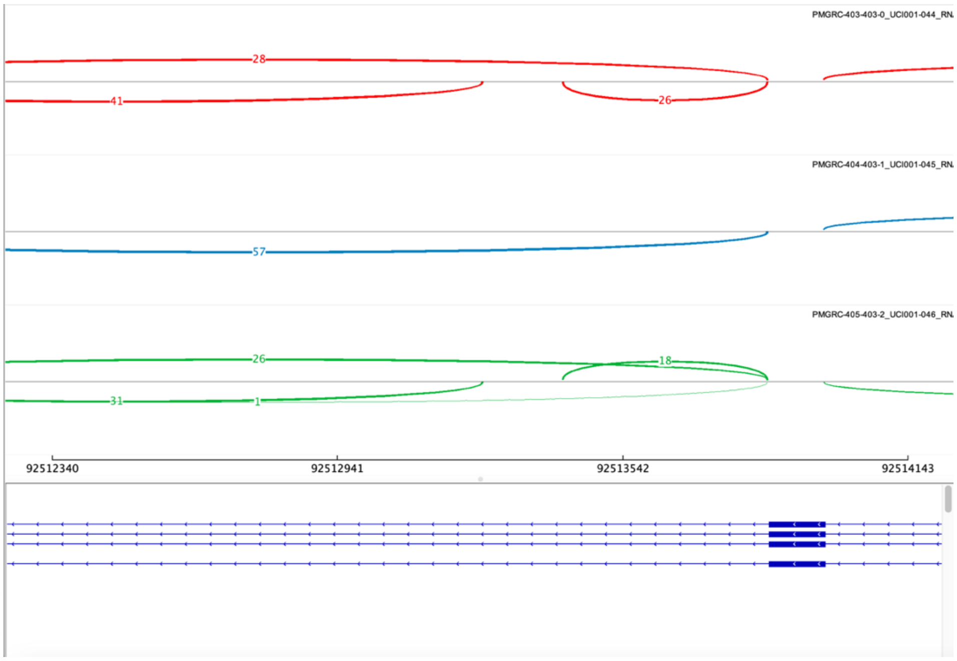

PMGRC-658-658-0

c.1282-4218G>A

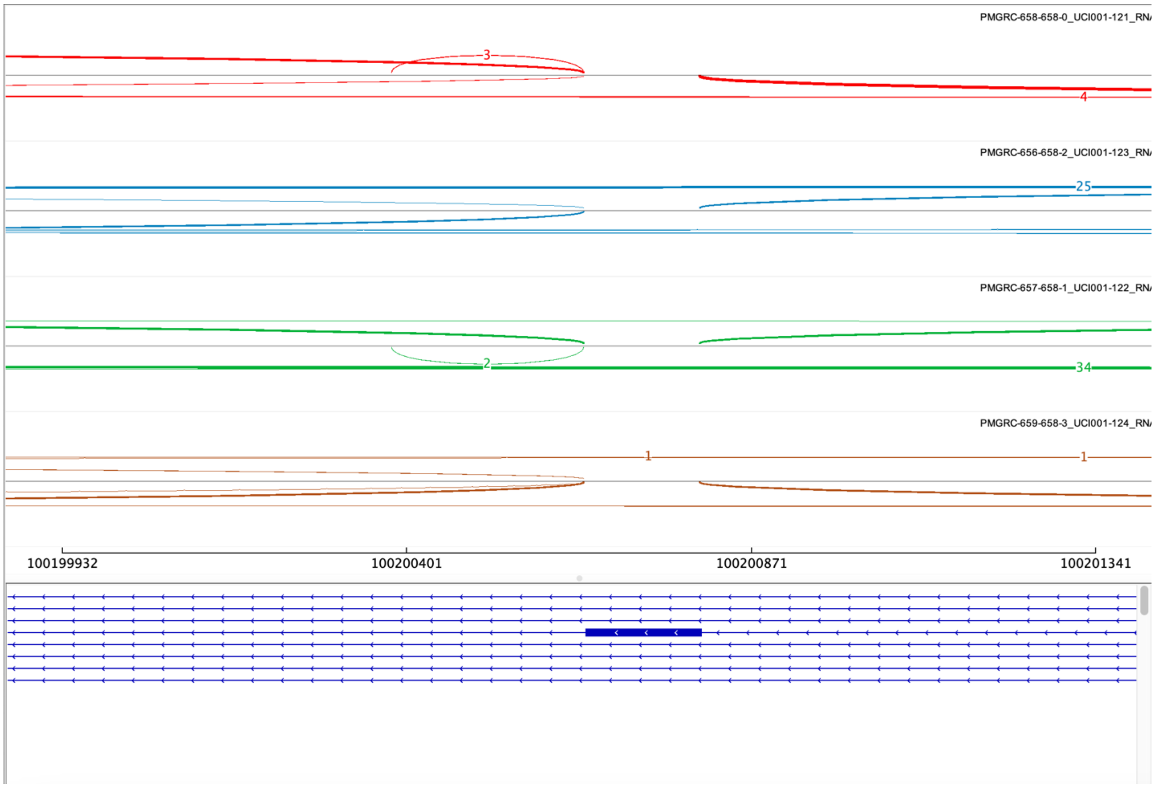

Supplemental Figure 2. Cryptic exon inclusions
